# Visualization of Polypropylene and Polyvinylidene Fluoride Slings in Perineal Ultrasound and Correlation with Clinical Outcome

**DOI:** 10.1155/2014/181035

**Published:** 2014-07-13

**Authors:** Laila Najjari, Julia Hennemann, Ruth Kirschner-Hermanns, Nicolai Maass, Thomas Papathemelis

**Affiliations:** ^1^Department of Gynaecology and Obstetrics, University Hospital Aachen, 52074 Aachen, Germany; ^2^Department of Urology, University Hospital Aachen, 52074 Aachen, Germany

## Abstract

*Introduction and Hypothesis.* Complications and malfunctioning after TOT can occur due to several factors, such as the material of the sling. The aim of the present study is to evaluate morphology and functionality of two types of slings (PVDF; polypropylene) *in vivo* using perineal ultrasound (PUS).* Materials*. In *n* = 47 women with TOT four criteria for PUS were taken and checked for possible differences: vertical stability of the sling position during Valsalva manoeuvre and contraction; distance “sling to urethra”; width of the sling and condition of the selvedges.* Results*. We observed an increased vertical displacement of the PP-slings, a significantly smaller variance to the extent of the displacement in PVDF-slings (*P* < 0.01), a significantly larger distance between sling and urethra (*P* < 0.001) in PVDF-slings, and a significantly smaller width of the PP-slings (*P* < 0.0001).* Conclusion*. Significant differences were found between the slings according to the four criteria. There was no difference established between the slings in the improvement of continence and no significant influence of the parameters was found for the resulting state of continence. In future studies, PUS may help to link differences in the morphology and functionality of *in vivo* slings to their material properties.

## 1. Introduction

Millions of women worldwide suffer from stress incontinence as the most frequent type of urinary incontinence. The huge impact of this illness on everyday life and social interaction necessitates an adequate treatment. During the last decade, a large variety of new operation techniques have been developed in order to achieve continence with as few complications as possible. The suburethral sling (tension-free vaginal tape, TVT, or transobturator tape, TOT) is one of the most promising approaches in this field. Even though it gives relief to many patients, in some cases the operation remains without success. Despite high initial success rates, at a 60-month follow-up, about 25% of all procedures end up in a relapse [1].

Some of the complications seen in TVT/TOT that may lead to relapse are different types of erosion: as reported in several studies, erosion of the vagina or the urinary tract can occur in 0.2 to 22% of the cases [2, 3]. It is described that PP causes an intense inflammation, whereas PVDF seems to be the synthetic material with the best biocompatibility, minimal foreign body reaction, and optimal ingrowth [4]. Bladder outlet obstruction and urge incontinence are further complications after TVT/TOT related to slings which are positioned too close to the bladder neck and to the urethra [5].

Incision, excision, and replacement of the slings are options of surgical intervention after the detection of sling complications. In order to facilitate the decision on the procedure, the exact etiology of the functional sling failure should be revealed. This is possible using perineal ultrasound, as seen in several studies [6–11].

There are some studies which have already been working on ultrasound evaluation of suburethral slings. Kociszewski et al. focused on the position of the tape in relation to the urethra [5]. However, they did not use the symphysis pubis as a fixed, bony reference point as proposed by Dietz et al. [8]; neither did they evaluate the dynamic properties of the sling position between rest and Valsalva manoeuvre, which might be an interesting aspect when thinking about material properties such as sling elasticity.

The shape of the slings in the midsagittal view has also been studied by Kociszewski et al., who concluded that tape functionality is given when it becomes C-shaped during Valsalva manoeuvre and stretches to a straight line during rest. The midsagittal shape of the sling is closely related to the width of the tape as a C-shaped sling is less wide than a straightly formed tape.

**Figure 1:**
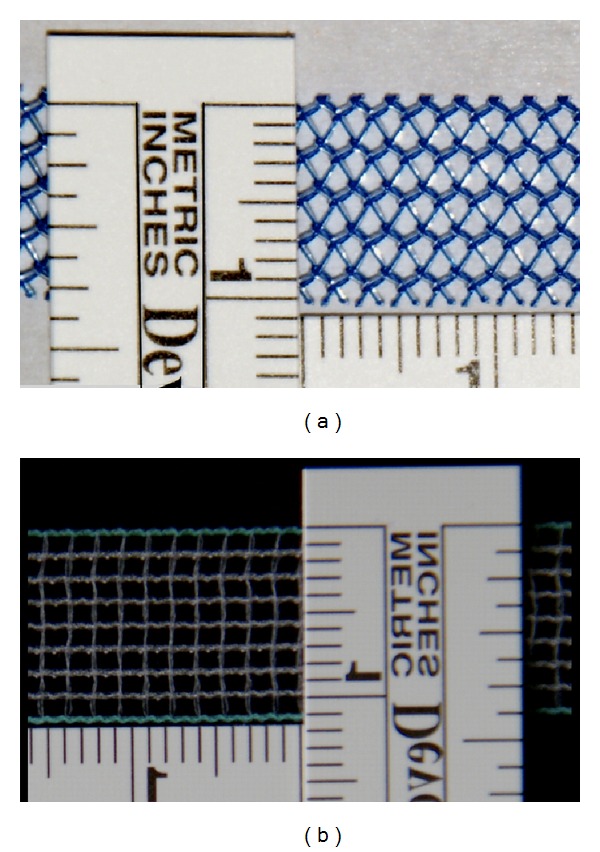
(a) A sling made of PP (GyneCare TVT, Ethicon). (b) A sling made of PVDF (DynaMesh SIS, Dahlhausen). Please note the differing textile structures of the slings.

Another interesting aspect is the state of the selvedges of the implanted sling. The word “selvedge” is defined as the edge of the sling. As seen in an* in vitro *study [12], it depends on the processing of the slings, if the selvedges are smooth and straight or sharply pointed (see Figure 1). This might also vary between rest and Valsalva manoeuvre.

In the present study, we will evaluate if material properties of suburethral slings can be assessed using perineal ultrasound and how they vary between different kinds of tapes. We will further study if their material properties have an influence on the resulting state of continence.

The patients evaluated in the present study had been implanted with two different kinds of slings: DynaMesh SIS, Dahlhausen, that is made of polyvinylidene fluoride (PVDF) and GyneCare TVT, Ethicon, that is made of polypropylene (PP). In our clinic, these are the most common materials for suburethral slings. They are produced by the extrusion of monofile filaments. Polypropylene is used as the basis for textile implants for almost five decades now [13], whereas PVDF has up to now been used for surgical hernia repair and is a novel material used in slings.

The present study is dedicated to several issues. First, we want to discuss if perineal ultrasound is able to evaluate material properties and structural behavior of slings* in vivo* and if the slings vary in their echogenicity. Second, we want to examine if there is a difference between the two types of slings concerning the resulting state of continence. Furthermore, we want to show if there is a difference between the above mentioned sling types by comparing the four parameters obtained by perineal ultrasound. These parameters are dynamic changes in the position of the sling between rest and Valsalva manoeuvre and between rest and pelvic floor contraction, distance between the sling and the anterior wall of the urethra, shape and width of the sling, and condition of the selvedges (sharply pointed versus smooth). At last, we want to discuss if these four characteristics have an influence on the resulting state of continence.

## 2. Materials and Methods

The present study is a retrospective observational study that was performed according to the Declaration of Helsinki and with the approval of the local ethics committee.

48 women (aged 60.7 years on average) were enrolled, who had all undergone a TOT-surgery for a clinically and urodynamically confirmed stress incontinence in our university hospital between April 2006 and February 2011. In 16 patients, a sling made of polyvinylidene fluoride (PVDF, DynaMesh SIS, Dahlhausen) had been implanted; the remaining 32 patients had been treated using a sling made of polypropylene (PP, GyneCare TVT, Ethicon); see Figure 1. All patients gave their informed consent.

All patients underwent perineal ultrasound evaluation and completed the ICIQ-SF questionnaire (International Consultation on Incontinence Modular Questionnaire-short form), an international validated instrument to assess incontinence, before and at least three months after the implantation of the sling. The operation had been performed 9 to 314 weeks, on average 104 weeks before the ultrasound evaluation was conducted.

In order to evaluate the sonographic visibility of these two types of slings irrespective of the surrounding tissue, an example of each sling was embedded in ballistic gelatine (Gelita AG, Gelatine type ballistic 3) in a 20% solution. After 30 min, the gelatine was heated in a 60°C bath and filled into a box containing the slings. For solidification, the box was cooled in a refrigerator at 8°C. Sonographic pictures of the slings were taken.

For objective evaluation of the implanted slings, we defined four criteria to describe the position and condition of the slings for sonographic measurement, partly borrowed from Klinge et al. and Kociszewski et al. [4, 5]. First, we examined the dynamic changes in the position of the sling between rest and Valsalva manoeuvre as well as between rest and pelvic floor contraction, performed on the patient in supine and upright positions. Second, we measured the distance between the sling and the anterior wall of the urethra. Third, we showed the shape and measured the width of the sling in the midsagittal view. Fourth and last, we described the condition of the selvedges (sharply pointed versus smooth).

**Figure 2:**
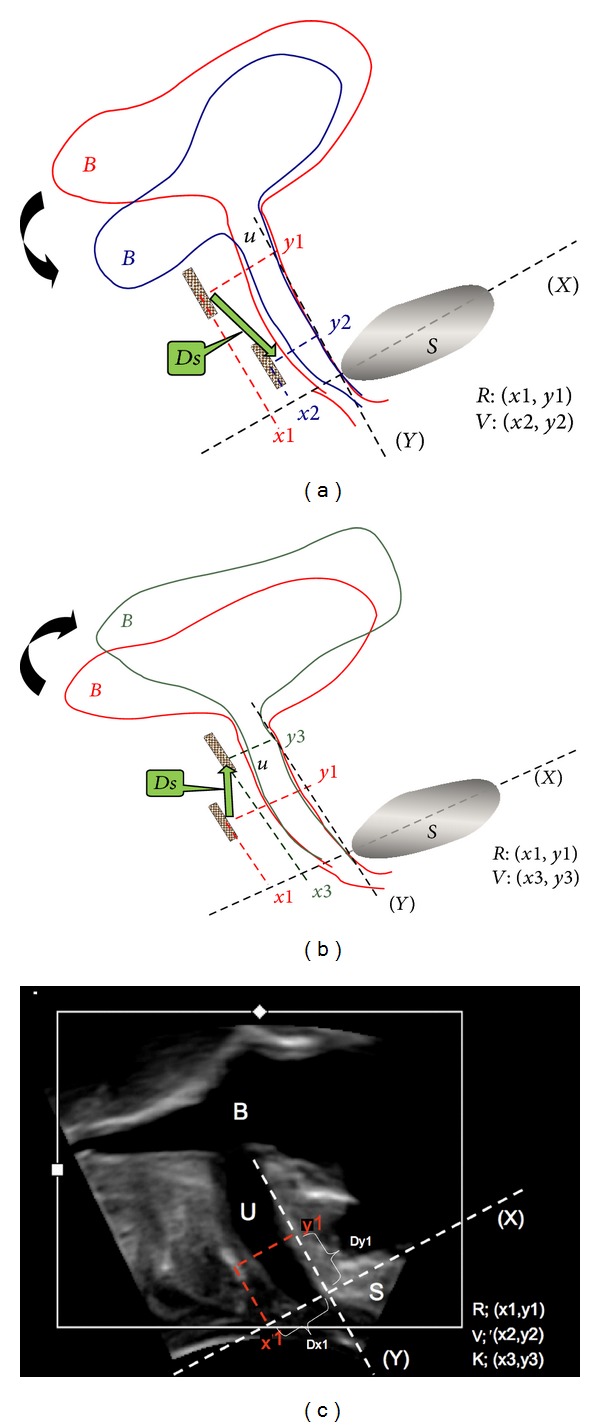
Measuring the sling position using a virtual coordinate system in perineal ultrasound, including symphysis (S), its central line equaling the *x*-axis (X), the *y*-axis, bladder (B), urethra (U), and the sling (brown coloured bar). (a) illustrates the movement of the lower urinary tract from resting state (red, coordinates *y*1/*x*1) to the position during straining (blue, *y*2/*x*2). The sling moves caudally along the green arrow (distance *Ds*). (b) presents the movement in the opposite direction (cranial) from resting state (red, position *y*1/*x*1) to the position during pelvic floor contraction (grey, position *y*3/*x*3). (c) is a picture taken from perineal ultrasound, applying the coordinate system. *Dx* is the distance from the sling to the anterior wall of the urethra (which equals the *x*-axis).

To find out whether the position of an implanted sling is robust along the vertical axis (i.e., along the urethra), its coordinates are measured during rest, Valsalva manoeuvre, and pelvic floor muscle contraction. For that purpose, we use a standardized coordinate system [14], as shown in Figure 2(a) to Figure 2(c). The distance *Ds* between the sling positions during rest (*x*1/*y*1, Figure 2(a)) and during Valsalva manoeuvre (*x*2/*y*2, Figure 2(a)), as well as *Ds* between the positions during rest (*x*1/*y*1, Figure 2(b)) and during pelvic floor contraction (*x*3/*y*3, Figure 2(b)), is calculated. This procedure is performed on the patient in both supine and upright positions.

The above mentioned standardized coordinate system is also applied to measure the distance *Dx* between sling and anterior wall of the urethra on the *x*-axis (Figure 2(c)). Furthermore, the width of the sling in the cross-sectional view can be measured. In “C-shaped” (curled-up) slings, we measured the distance between the two arms of the “C.” In sonographic evaluation, we defined the selvedges of a sling which can be seen as smooth or as sharply pointed.

All perineal ultrasound (PUS) evaluations were performed by one single examiner who is qualified according to the DEGUM II standard. The data were obtained in standing and lithotomy positions after the patients had drunk about 350 mL of water, resulting in a bladder filling of 250–300 mL. Ultrasound assessment was carried out in the midsagittal plane using a 3,5-5 MHz perineal transducer (Voluson 730 expert, GE Kretz, Ultrasound, Zipf, Austria), allowing an acquisition angle of 70°. The evaluation of the ultrasound pictures was performed by two independent examiners without any knowledge of the sling material used.

Continuous variables are expressed as mean values ± standard deviation. To compare vertical movement of slings from resting position, distance from the anterior wall of the urethra to the sling, and width of the slings, all between PVDF and PP, the two-sided nonparametric Wilcoxon rank sum test for independent samples was conducted. Variance of sling dislocation from resting position was investigated by the nonparametric Ansari-Bradley test.

ICIQ was assessed at baseline and after intervention. Analysis of covariance (ANCOVA) was carried out in order to investigate the influence of material on state of continence.

Correlation analyses investigated the association of ICIQ-difference and various parameters of interest. Spearman's rank and Pearson's correlation coefficient were determined to assess monotonic and linear trends.

Count data are presented by frequencies and percentage. Count data was evaluated using Fisher's exact test.

Significance was assigned at *P* ≤ 0.05. Data were analyzed using R (R Version 2.11.1., Copyright (C) 2010, The R Foundation for Statistical Computing).

## 3. Results

Our study yielded the following results.

The evaluation of the slings was feasible in 48 patients. Because of a defect in the data set of one patient, only 47 data sets were analyzed. 16 patients got a PVDF-sling and 32 patients got a PP-sling.

**Figure 3:**
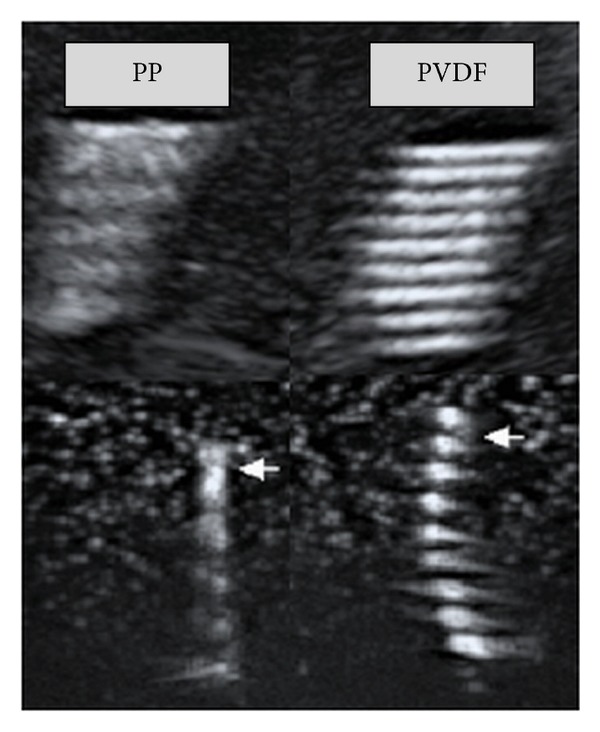
Sonography of both kinds of slings embedded in gelantine, performed to compare their individual sonographic visibility. Upper row: longitudinal aspect; lower row: cross-sectional aspect.

The sonographic evaluation of the slings embedded in gelatine revealed that both slings were equally visible (see Figure 3).

During Valsalva manoeuvre as well as during contraction, we observed a slightly increased displacement of the PP-slings. This was more obvious in the upright positioned patient. However, the difference was statistically significant only during contraction at the standing patient. Furthermore, the values of maximal descent of the slings were larger (see Table 1 for detailed values).

To find out if the slings made of the same material (either PP or PVDF) have a similar stability, the variance of the descent was measured and averaged. The Ansari-Bradley test revealed that PVDF-slings have a significantly smaller variance concerning the extent of their displacement only during straining in the upright positioned patient. In all other conditions, there was no significant difference between the slings (see Table 2).

We found that the distance *Dx* (as seen in Figure 2(c)) from the sling to the anterior wall of the urethra tended to be larger in PVDF-slings than in PP-slings. This difference, however, was statistically not significant (see Table 3).

**Figure 4:**
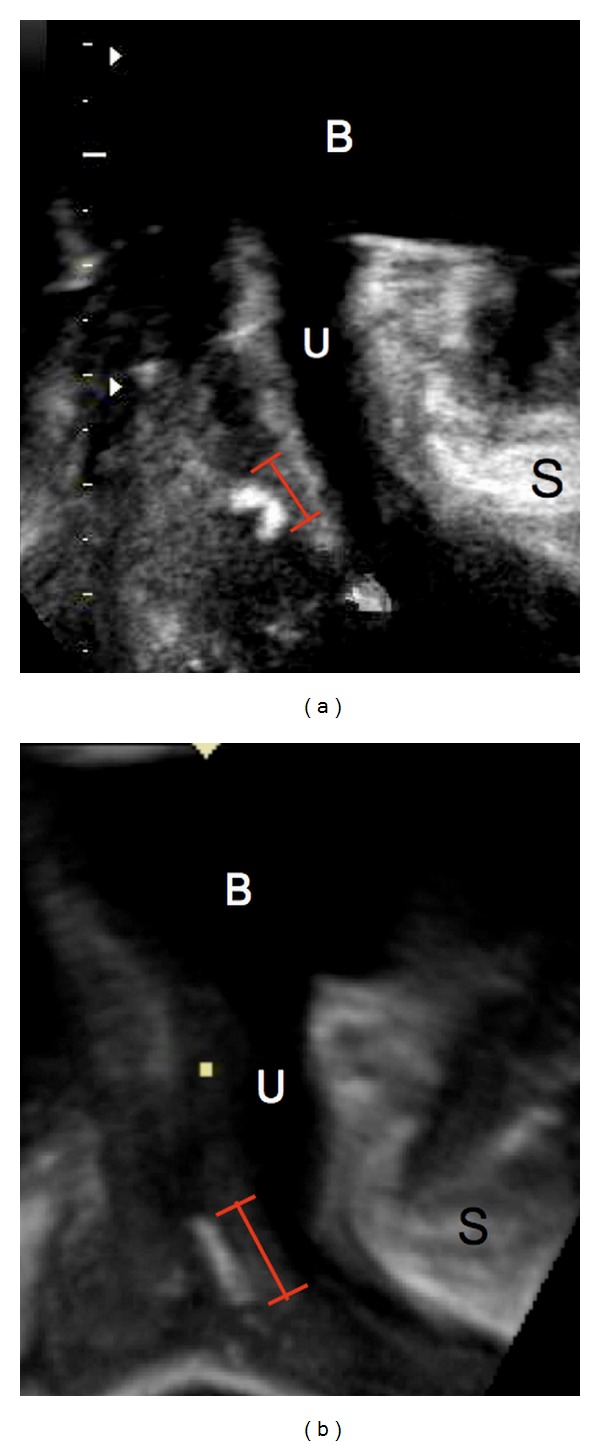
(a) A rolled-in PP-sling with smaller effective width (red labeling). (b) A straight PVDF-sling with regular width. Also included are bladder (B), urethra (U), and symphysis (S).

12.5% of all PVDF-slings and 78.1% of all PP-slings were curled up (Figure 4(a)). The remaining slings were in straight configuration (Figure 4(b)). Fisher's exact test for count data revealed a significant difference (*P* < 0.0001).

To assess the width of curled-up slings, the distance between the two arms of the “C”-shaped sling, implying the proportion of the sling with contact to the urethra, was measured. The mean width of PVDF-slings was 0.79 cm (SD 0.23) and that of PP-slings was 0.62 cm (SD 0.23), proving a significantly smaller width of the PP-slings compared to the PVDF-slings (*P* < 0.05).

**Figure 5:**
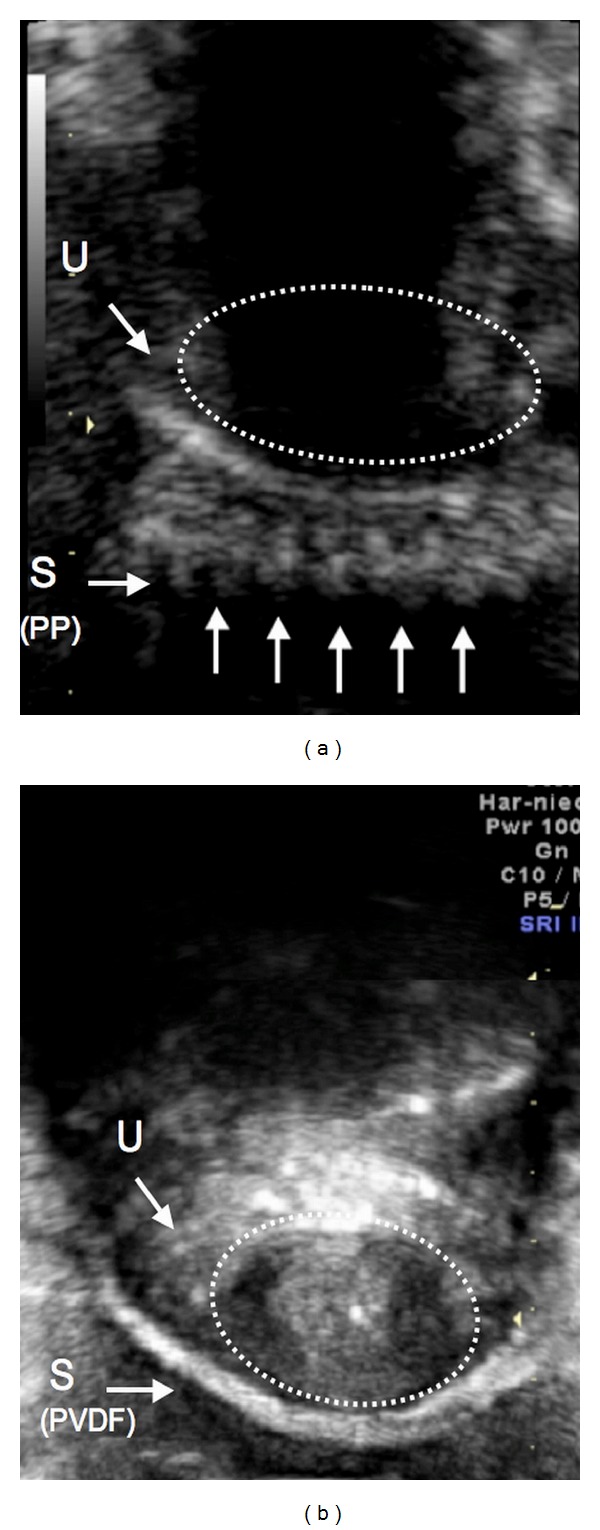
Picture from perineal ultrasound showing a PP-sling “S (PP)” with sharp pointing selvedges (arrows, (a)). A PVDF-sling “S (PVDF)” with smooth selvedges (b). Inside the dotted line: urethra (U).

In all PP-slings, sharp pointing selvedges were observed as seen in Figure 5(a) (arrows), whereas all PVDF-slings had smooth selvedges (Figure 5(b)).

Table 4 shows the respective ICIQ result before and after the intervention (0 = no urine loss; 21 = maximum urine loss and interference with everyday life).

Even if there is a trend towards a better surgical result with PVDF-slings, the analysis of covariance did not reveal any significant difference between both types.

For the correlation between the improvement in the ICIQ value and stability of the sling, there was no correlation during contraction and there was only poor correlation during Valsalva manoeuvre. Concerning the distance between sling and urethra, poor to very poor correlation was found. The parameter with the highest (but still poor) correlation with the clinical outcome was the distance from sling to urethra at rest in upright position. For detailed values, see Table 5.

There was no correlation between the width of the slings and the ICIQ development.

Curling-up of the slings does not seem to have an influence on the resulting state of continence development, as the mean values of ICIQ difference are very similar (not curled: 9.18; curled: 9.22).

The presence of rough or smooth sling edges does not seem to influence the resulting state of continence: all PVDF-slings had smooth edges, all PP-slings had rough edges, and between the two sling types there was no difference as to the outcome (see Table 4).

## 4. Discussion

The results of the present study demonstrate that perineal ultrasound allows to visualize tension-free vaginal slings and to objectively establish their position and morphology in the pelvis.

The sonographic evaluation of the two slings embedded in gelatine revealed that their visibility via ultrasound is equal, which is mandatory to compare their properties* in vivo*.

Since sonography is a dynamic procedure, which considerably depends on the examiner, we defined objective criteria for an examiner-independent evaluation. These are given as follows: stability of the sling position during Valsalva manoeuvre and pelvic floor muscle contraction, distance between the sling and the urethra, width of the sling, and condition of the selvedges (sharply pointed versus smooth). These parameters allowed the characterization of the slings in all patients.

Concerning these four criteria, the slings revealed differing characteristics.

For the extent of sling dislocation, there were only slight differences between the two types of slings with a trend for PVDF-slings to move a little less. This is consistent with the findings of Müllen and Göretzlehner, who determined the elasticity of PP-slings and PVDF-slings to be 47% and 7%, respectively [12]. Particularly during contraction in the upright position, PP-slings moved significantly more in the cranial direction than PVDF-slings. Concerning the improvement of the ICIQ value, sling dislocation does not seem to have any significant influence, even if there is a trend towards a better clinical outcome with greater displacement.

Our results show that among PP-slings, there is a significantly greater variation with regard to the displacement than among PVDF-slings, but only during straining in the upright position. This is possibly the consequence of a much more elastic sling [12], which is pushed far downwards by a strong straining and descends less during a weak straining.

Concerning the distance between sling and urethra, our data are heterogeneous. In general, there are only minimal differences between the slings, except during Valsalva manoeuvre in the standing position. Here, PP-slings were much closer to the urethra than PVDF-slings.

Values are smaller in the standing position, telling that the sling is possibly being pressed against the urethra by the weight of the organs above.

A sling positioned too close to the urethra may result in obstructive complications [5]; an adequate distance between urethra and sling can therefore be beneficial to the functionality of the sling and is even intended for a “tension-free” tape. Kociszewski et al. report an optimal sling-urethra distance of 3–5 mm at a 6-month follow-up [5]. Unfortunately, the authors did not report if they took the distance during rest or straining. For the Valsalva manoeuvre, our results are in agreement with their proposal, except for PP-slings during straining, being 1.3 mm away from the urethra. Following Kociszewski et al., obstructive complications are much more common if the distance is less than 2 mm [5].

Our data do not reveal any important impact of the distance between sling and urethra on the clinical outcome. There was, however, a trend towards better results with a larger distance, which is consistent with the findings of the above mentioned authors.

In general, it was feasible to measure the width of the slings at rest. Due to a high rate of curled-up slings among the PP-slings (78%) in contrast to the PVDF-slings (12%), we focused on the “effective width” of the sling, that is, the part of the sling with direct contact to the urethra. In rolled-in slings, this was established by measuring the distance between the two arms of the C-shaped sling, whereas in straight slings the effective width was equal to the actual width. Naturally, the effective width of curled-up slings is inferior to the one of straightly configured slings, so we observed a significantly smaller effective width in PP-slings in comparison to PVDF-slings. Our results did not confirm any influence of the width on the clinical outcome.

Müllen and Göretzlehner confirmed the tendency of the PP-slings to roll in in an* ex vivo* study [12], which suggests that rolling-in is a material- and structure-dependent characteristic. Kociszewski et al. also observed C-shaped slings and concluded that rolling-in during Valsalva manoeuvre, but not at rest, is a proof for sling functionality [5]. Slings, which were curled up already at rest, showed higher rates of recurrence, urge, and voiding difficulty [5]. Curling-up also results in a reduced pore size, as Müllen and Göretzlehner confirmed in an* ex vivo* study [12]. Following Slack et al., this might be associated with a stronger inflammatory reaction of the surrounding tissue [15]. Our outcome does not confirm any influence of curled-up slings on the resulting state of continence.

In the present study, in all PP-slings, sharply pointing selvedges were seen, whereas, in all PVDF-slings, smooth selvedges without those fibers were observed (Figure 5). As seen in Figure 1, this is probably due to a different structure of the slings. The presence of open selvedges affects the structural stability of the sling during straining [12], which might explain why PP-slings are found to be curled up more than eight times as often as PVDF-slings. This is also a possible reason for a much greater elasticity as established by Müllen and Göretzlehner [12]. Sharply pointing fibers are discussed to stimulate chronic inflammatory processes [4]. In our study, we could not detect any influence of the state of the selvedges on the resulting state of continence.

We conclude that the properties of the slings evaluated in this study suggest that already, in a neutral position at rest, PP-slings seem to lose their structural integrity by curling-up and decreasing in width, which seems rather discrete in PVDF-slings. For the clinical outcome, curling does not seem to cause any difference.

The average result of the ICIQ questionnaire before the intervention was nearly the same in PP- and PVDF-slings, confirming a homogeneous sample of patients. Implantation of the slings reduced the score by 9.82 points in PVDF-slings and by 9.09 points in PP-slings, which, in both cases, is an acceptable surgical result. There seems to be a trend towards a better result with PVDF-slings. This difference, however, is not significant, which might be due to the small sample size. Another result of the outcome analysis is a huge standard deviation for both slings after the intervention, which possibly mirrors a heterogeneous surgical result.

The association between the clinical outcome and the parameters found in this study was—except for the distance between sling and urethra—a first approach and should be taken with care, as our sample size is too small to make a clear statement. It should be a goal for the future to relate complications and malfunctioning to certain sonographic properties.

In summary, perineal ultrasound emerges as the ideal examination technique for a long-term assessment of sling implants. A concise definition of sonographic criteria supports the objective estimation of morphologic and functional findings. Comparing the two types of slings of differing structure and material (PP and PVDF), differences in form and dynamics could be detected, especially the tendency of PP-slings to curl up. Concerning the analysis of the clinical outcome, represented by the ICIQ score, differences between these slings were statistically not significant. We observed a slight trend towards a better surgical result with increasing distance from sling to urethra and increasing displacement of the sling during straining. However, none of the parameters correlated considerably with the clinical outcome.

## Figures and Tables

**Table 1:** Vertical movement of the slings from resting position during contraction and Valsalva manoeuvre (in cm).

	Mean (SD)	Min.	Max.	*P* value
Supine position				
Contraction				
PVDF	0.09 (±0.07)	0.01	0.21	0.30
PP	0.12 (±0.06)	0.02	0.27	
Valsalva				
PVDF	0.25 (±0.15)	0.09	0.64	0.96
PP	0.29 (±0.26)	0.02	1.34	

Upright position				
Contraction				
PVDF	0.08 (±0.06)	0.01	0.18	<0.05
PP	0.19 (±0.19)	0.01	0.75	
Valsalva				
PVDF	0.13 (±0.07)	0.04	0.24	0.11
PP	0.24 (±0.20)	0.02	0.77	

SD: standard deviation; Min.: minimum value; Max.: maximum value; PVDF: polyvinylidene fluoride; PP: polypropylene.

**Table 2:** Variances of sling dislocation from resting position during contraction and Valsalva manoeuvre, compared for PP and PVDF by the Ansari-Bradleytest.

Position	Manoeuvre	Variance, PVDF (cm^2^)	Variance, PP (cm^2^)	*P* value
Supine position	Contraction	0.005	0.004	0.81
	Valsalva	0.023	0.068	0.08

Upright position	Contraction	0.004	0.036	0.85
	Valsalva	0.005	0.040	<0.03

**Table 3:** Distance from the anterior wall of the urethra to the sling in cm.

	Mean (SD)	Min.	Max.	*P *value
Supine position				
Rest				
PVDF	1.05 (±0.37)	0.55	2.05	0.27
PP	0.89 (±0.23)	0.45	1.6	
Contraction				
PVDF	0.99 (±0.31)	0.61	1.6	0.14
PP	0.82 (±0.21)	0.32	1.27	
Valsalva				
PVDF	1.04 (±0.43)	0.55	2.29	0.22
PP	0.86 (±0.25)	0.4	1.44	

Upright position				
Rest				
PVDF	0.94 (±0.25)	0.63	1.4	0.21
PP	0.83 (±0.19)	0.48	1.31	
Contraction				
PVDF	0.89 (±0.26)	0.55	1.67	0.13
PP	0.79 (±0.21)	0.45	1.39	
Valsalva				
PVDF	0.89 (±0.26)	0.35	1.35	0.12
PP	0.78 (±0.23)	0.4	1.36	

SD: standard deviation; Min.: minimum value; Max.: maximum value; PVDF: polyvinylidene fluoride; PP: polypropylene.

**Table 4:** Differences in ICIQ results before and after sling implantation: mean (±SD).

	Before sling	After sling
PVDF	15.4 (±4.3)	5.6 (±5.9)
PP	15.3 (±3.4)	6.2 (±6.6)

**Table 5:** Correlation between sling stability, distance from tape to urethra, and width of the sling and improvement in ICIQ value.

	Sling stability		Distance from sling to urethra	
	Pearson	Spearman	Pearson	Spearman
Supine position				
Contraction	−0.02	−0.03	0.16	0.23
Valsalva	0.22	0.18	0.18	0.18
Rest	—	—	0.19	0.18

Upright position				
Contraction	0.04	0.08	0.31	0.31
Valsalva	0.23	0.25	0.12	0.10
Rest	—	—	0.35	0.42

Width	−0.04	−0.03		
